# Depressive Symptoms in People with and without Alcohol Abuse: Factor Structure and Measurement Invariance of the Beck Depression Inventory (BDI-II) Across Groups

**DOI:** 10.1371/journal.pone.0088321

**Published:** 2014-02-12

**Authors:** Cecilie Skule, Pål Ulleberg, Hilde Dallavara Lending, Torkil Berge, Jens Egeland, Tim Brennen, Nils Inge Landrø

**Affiliations:** 1 Community Mental Health Center Vinderen, Diakonhjemmet Hospital, Oslo, Norway; 2 Department of Psychology, University of Oslo, Oslo, Norway; 3 Vestfold Mental Health Care Trust, Tønsberg, Norway; McGill University, Canada

## Abstract

This study explored differences in the factor structure of depressive symptoms in patients with and without alcohol abuse, and differences in the severity of depressive symptoms between the two groups. In a sample of 358 patients without alcohol problems and 167 patients with comorbid alcohol problems, confirmatory factor analysis revealed that the same factor structures, Beck et al.'s two-factor Somatic Affective-Cognitive (SA-C) model, and Buckley et al.'s three-factor Cognitive-Affective- Somatic (C-A-S) model, demonstrated the best fit to the data in both groups. The SA-C model was preferred due to its more parsimonious nature. Evidence for strict measurement invariance across the two groups for the SA-C model was found. MIMIC (multiple-indicator-multiple-cause) modeling showed that the level of depressive symptoms was found to be highest on both factors in the group with comorbid alcohol problems. The magnitude of the differences in latent mean scores suggested a moderate difference in the level of depressive symptoms between the two groups. It is argued that patients with comorbid depression and alcohol abuse should be offered parallel and adequate treatment for both conditions.

## Introduction

The co-occurrence of mood symptoms and the abuse of alcohol and other drugs is common [1], [2], [3]. Patients with comorbid major depression and substance abuse tend to be more severely depressed than those without the combined condition [4]. Among medical inpatients, more severe depressive symptoms are associated with unhealthy drinking [5].

Self-report instruments, such as the Beck Depression Inventory-II (BDI-II), are often used in clinical practice and research. However, research on the BDI-II and other self-report instruments in patients with comorbid depression and substance use disorders is scarce [6]. Studies have investigated the factor structure of the BDI-II [8], [9]. A three-factor model consisting of a cognitive, affective and somatic factor seems to usually give the best fit [10], [11], [12], but there are exceptions [7], [9]. It has been claimed that the factor structure of BDI-II scores differ among various clinical populations [13]. If the BDI-II measure fails to operate in the same manner across groups (i.e. there is a lack of measurement invariance of the construct), the between-group differences in the mean scores of the BDI-II may be misleading. However, no previous study has compared the severity of depressive symptoms and the factor structure of depressive symptoms between a clinical sample of depressed participants with and without comorbid alcohol abuse.

It is often assumed that the clinical implications of depressive symptoms in patients with substance abuse are different than those in depressed patients without substance abuse. This study examines the factor structure of the BDI-II in a large clinical sample of people seeking treatment for depression. Some of the participants have concurrent problems of alcohol abuse, therefore allowing a comparison of the two samples.

In this study we have two research aims: to identify differences in the factor structure of depressive symptoms in patients with and without alcohol abuse, and to identify differences in the severity of depressive symptoms between the two groups.

## Subjects and Methods

### Participants

Participants were recruited from the attendees of cognitive-behavioral courses of treatment for depression either via individual consultations or in groups. In the preconsultation or during information meetings held by group leaders/therapists conducted before treatment start, the participants were informed about the study and asked if they would like to participate in this research project. They were informed that participation was voluntary and were informed that they could leave the project at any time, and if they decided not to participate in the project, it would not have any consequences for the treatment they were offered. Participation in the project was not paid for. Exclusion criteria for taking part in the treatment program were psychotic or acute suicidal symptoms. The participants were from the same health region in the south and east part of Norway. Most of the participants were recruited from community mental health centers. A small group was recruited from a substance abuse clinic. In total 525 patients provided written informed consent, and all participants were considered to possess competent consent. The project was approved by Regional Committees for Medical and Health Research Ethics in the South East Health Region in Norway. The research was funded by the Regional Competence Centre for Double Diagnoses, South Eastern Norway and the research fund in the Community Mental Health Center, Vinderen, Diakonhjemmet Hospital, Oslo. The funders had no role in study design, data collection and analysis, decision to publish, or preparation of the manuscript.

### Instruments

*The Beck Depression Inventory* - *Second Edition* (BDI-II) [7] is one of the most commonly used self-report instruments for estimating the severity of depression. The total score indicates whether the individual presents a mild, moderate or major depression. The BDI-II consists of 21 items, each of which is scored on a scale from 0 to 3. The maximum score is 63. The recommended cutoff for minimal depression is 13, whereas a score of 14–19 indicates mild, 20–28 moderate and 29–63 serious depression.

*The Alcohol Use Disorders Identification Test* (AUDIT) [14] consists of ten items and can be self-administered by the patient. Each item is scored on a 4-point scale. The total ranges from 0–40, and a score larger than 7 indicates an alcohol problem. Based on research the following categories have been identified for the total score: 0–7 low risk, 8–15 moderate risk and 16–19 major risk.

### Procedure

A total of 525 patients provided informed consent. In addition to the BDI-II and substance abuse screening using the AUDIT, participants answered questions on their demographics and their history of depression. The patients completed the screening before the cognitive-behavioral course of treatment for depression started.

### Statistical Analyses

Confirmatory factor analyses based upon maximum likelihood estimation were applied to test the fit of the various models to the data of both samples (descriptive statistics and intercorrelations among the 21 items is presented separately for each sample in Appendix A and B). All models were estimated using the statistical software Mplus 6.1 [15]. Because the data were expected to have a non-normal distribution, the models were estimated using maximum likelihood estimation and tested with the Satorra-Bentler scaled chi-square [16]. The fit of the models was evaluated using several *χ*2 goodness-of fit-statistics; the comparative fit index (CFI), the root mean square error of approximation (RMSEA) and the standardized root mean squared residual (SRMR). As a general rule, a CFI above .95, and a RMSEA/SRMR below .06 indicates a very good fit between the model and the data, whereas a RMSEA below .08, SRMR below .09 and a CFI above .90 is conventionally regarded as a reasonable fit [17], [18]. The fit of the various factor structures were estimated separately for each sample.

Measurement invariance across groups of the best-fitting factor structures was tested in several models, using Multigroup Confirmatory Factor Analysis. The procedure involved examining changes in model fit measures after imposing increasingly restrictive conditions of invariance [19]. The first model tested whether the same items are associated with the same factor in both groups. This is commonly referred to as configural invariance. The second model involved examining the invariance of the factor loadings, meaning that the loadings are the equal in both groups. The equality of covariance between residuals was also included in this model. The third model tested for invariance at the intercept level of the items. This level of invariance (together with measurement invariance established in the previous models) is required to compare differences in the latent mean scores between groups. The fourth model involved invariance of the item residuals, signifying that all group differences on the items are due to group differences on the level of the common factors. This model is, however, regarded as representing a very stringent criterion, and is rarely fulfilled in practice [20], [21]. Two additional models were also estimated; model 5 tested for invariance in factor variances and model 6 for invariance in the covariance between factors across groups.

Traditionally, evidence supporting measurement invariance has been based on non-significant differences in the chi-square value (Δ*χ*2) relative to the change in the degrees of freedom (Δ*df*) between nested models. If the Δ*χ*2 is significant for the more restrictive model, it can be assumed that that the two models are not equivalent (i.e. non-invariant) across groups. However, the Δ*χ*2 value is highly sensitive to the sample size, and based on simulation studies, researchers [20], [21] have recommended alternative criteria for evaluating measurement invariance across groups. According to Chen [21], the following cutoff values have been suggested to indicate non-invariance in large samples (N>300): a change of ≥−.01 in CFI, supplemented by a change of ≥.015 in RMSEA or a change of ≥.030 in SRMR.

Differences in the severity of depressive symptoms between the groups with and without comorbid alcohol problems was examined using MIMIC (multiple-indicator-multiple-cause) modeling. MIMIC modeling is comparable to a multivariate regression model in which latent variables (e.g. latent scores on factors of depression) are “caused” by independent variables. The main independent variable in this case the grouping variable separating between those with and without comorbid alcohol problems. Possible systematic differences between the two groups on other variables potentially related to depression scores (e.g. gender, age) may also be included in such an analysis as independent variables and thereby serve as covariates.

## Results

### Descriptive Statistics

Table 1 shows descriptive statistics for both samples. There were no significant differences in age and educational level between the two samples. There were significantly more men among the participants with a comorbid alcohol problem, and they were less likely to be married or have a registered partner compared to the sample without comorbid alcohol problems. The level of depression (as measured by the total BDI-II score) was 3.5 points higher in the sample with comorbid alcohol problems.

**Table 1:** *Patient demographics, AUDIT-score, and BDI-II-score.*

	*Without comorbid alcohol problem (n = 358)*	*With comorbid alcohol problem (n = 167)*	*t or χ^2^-value*
Age (*M*, *SD*)	42.2 (11.3)	41.8 (13.0)	0.37
% Men	29.5	50.3	
% Women	70.5	49.7	22.09***
Highest educational level attained			
% Lower secondary	7.4	6.1	
% Upper secondary/vocational	11.6	9.8	
% Upper secondary/academic	13.3	20.2	
% Tertiary	67.7	63.8	4.57
Marital status			
% Single	29.1	39.0	
% Married/reg. partner	57.1	37.2	
% Divorced	12.4	23.2	
% Widow/widower	1.4	0.6	20.9***
AUDIT-score (*M*, *SD*)	3.2 (2.0)	14.5 (5.8)	−32.0***
Total BDI-II-score (*M*, *SD*)	23.1 (11.1)	26.6 (10.1)	−3.40***

*** *p*<.001.

### Model Testing

**Figure 1:**
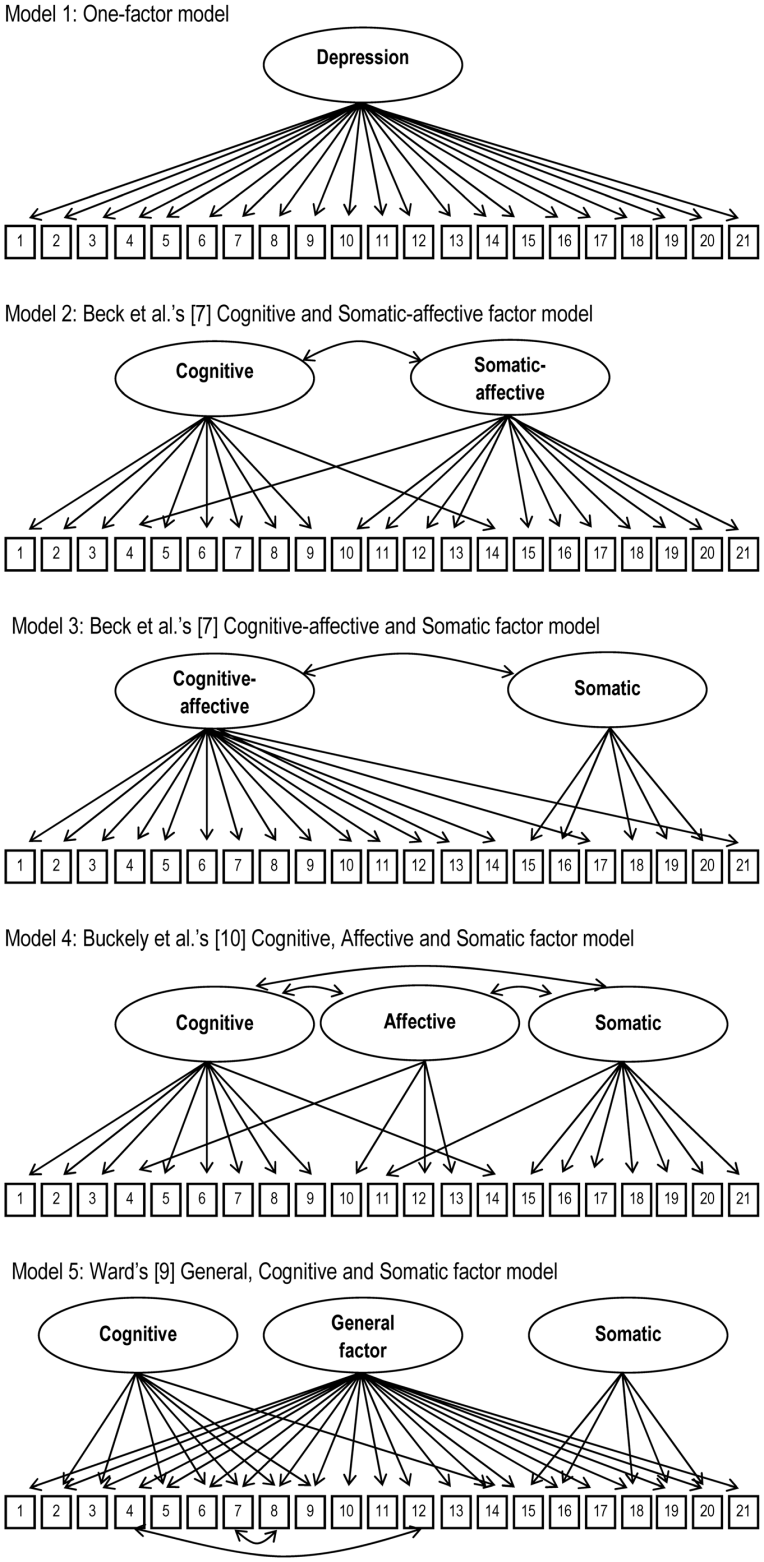
Factor structure of the different models tested.

The alternative models presented in Figure 1 were separately estimated for each sample using confirmatory factor analysis. Table 2 shows that all models had good or acceptable RMSEA/SRMR values in both samples. In the sample without comorbid alcohol problems, the CFI for the one-factor model and the Beck et al.'s [7] CA-S model was below the conventional limits for acceptable fit (>.90). In the sample with concurrent alcohol problems, only the model proposed by Ward [9] had a CFI value above the threshold for acceptable fit.

**Table 2:** *Confirmatory factor analysis fit indices for five different factor models. Separately for the samples with and without comorbid alcohol problems.*

	*SB χ^2^*	df	RMSEA	SRMR	CFI
**Without comorbid alcohol problem (** ***n*** ** = 358)**					
*Model 1: One factor model*	557.6	189	.074	.056	.867
*Modified model* b	396.4	187	.056	.049	.919
*Model 2: Beck et al. * [*7*] * SA-C*	431.1	188	.060	.048	.936
*Modified model* b	312.0	186	.043	.042	.950
*Model 3: Beck et al. * [*7*] * CA-S*	464.1	188	.064	.054	.893
*Modified model* b	360.7	186	.051	.046	.932
*Model 4: Buckley et al. * [*10*] * C-A-S*	399.4	186	.057	.047	.917
*Modified model* b	299.1	184	.042	.040	.955
*Model 5: Ward * [*9*] * G-C-S* a	306.7	174	.046	.041	.949
**With comorbid alcohol problem (** ***n*** ** = 167)**					
*Model 1: One factor model*	363.5	189	.074	.069	.836
*Modified model* b	331.7	187	.068	.067	.864
*Model 2: Beck et al. * [*7*] * SA-C*	335.1	188	.068	.069	.862
*Modified model* b	311.1	186	.063	.067	.882
*Model 3: Beck et al * [*7*] * CA-S*	339.1	188	.069	.070	.858
*Modified model* b	325.5	186	.067	.067	.869
*Model 4: Buckley et al * [*10*] * C-A-S*	316.7	186	.065	.067	.877
*Modified model* b	301.0	184	.062	.066	.890
*Model 5: Ward * [*9*] * G-C-S* a *^, ^* c	261.9	175	.055	.061	.918

*SB χ^2^* = Satorra-Bentler corrected *χ^2^*; *df* = degrees of freedom; RMSEA = root mean square of approximation; SRMR = Standardized root mean square; CFI = comparative fit index.

a Correlated residuals allowed for Items 4–12 and 7–8,

b Correlated residuals allowed for Items 5–8 and 15–20.

c Residual variance of item 20 constrained to zero.

The model formulated by Ward [9] was therefore the only one able to fulfill all the criteria for good or acceptable fit in both samples. However, a closer inspection of the factor loadings in Ward's model revealed the cognitive factor had several non-significant loadings in both samples. The proposed error covariance between Item 7 and Item 8 in the sample with concurrent alcohol problems was also nonsignificant. Furthermore, the residual variance was negative for item 20 and had to be constrained to zero to allow the model to converge. This feature suggested that Ward's [9] model was problematic due to inadequate factor loadings, and was therefore abandoned from further analyses. This justify studying the modification indices of the alternative models more closely. In all alternative models, the modification indices indicated that a rather large reduction in *SB*-chi square value could be obtained by allowing two pairs of correlated residuals: the first between Item 5 (guilty feelings) and Item 8 (self-criticalness): and the second between Item 15 (loss of energy) and item 20 (tiredness or fatigue). Both modifications were regarded as theoretically meaningful, because the items semantically overlapped and both pair of items clustered on the same factor in all models. No further modifications were found necessary.

Table 2 shows that the fit indices for all models improved after the modifications were implemented. In the sample without concurrent alcohol problems, all models demonstrated an acceptable or good fit to the data. In particular, Beck et al.'s [7] SA-C model and Buckley et al.'s [10] C-A-S model were regarded as better-fitting than the CA-S and one-factor model due to the difference in CFI-value (>.01) between these models. Although a Satorra-Bentler scaled chi-square difference test demonstrated that the Buckley et al. [10] model fitted significantly better to the data than the Beck et al. [7] SA-C model (SB *χ* ^2^_diff_ (2) = 13.2, *p*<.01), this difference was regarded as trivial due to the small differences in model fit measures between the two models.

In the sample with comorbid alcohol problems, Beck et al.'s [7] SA-C model and Buckley et al.'s [10] C-A-S model also demonstrated the best fit to the data compared to the two other modified models (e.g. ΔCFI≥.01). However, only the RMSEA and SRMR suggested adequate fit to the data for the two models, whereas the GFI and CFI were close to, but did not reach the threshold for acceptable values. Buckley et al.'s [10] model was significantly better fitting than Beck et al.'s [7] SA-C (SB *χ* ^2^_diff_ (2) = 10.3, *p*<.01) in this sample also. However, the comparison of the other model fit indices suggested that the difference between the two models was small (e.g. ΔCFI<.01).

Based on the small difference in model fit indices between the two models, Beck et al.'s [7] SA-C model was chosen as the preferred one due to its more parsimonious nature, i.e. represented by two factors instead of three factors as in the C-A-S model [10].

### Measurement Invariance across Samples

Beck et al. [7] SA-C model was tested for measurement invariance across the two samples. As shown in Table 3, the change in fit indices fell well below the recommended cutoff values (ΔRMSEA≥.015, ΔCFI≥−.01 and ΔSRMR≥.030) at all six steps for testing invariance. Therefore, the results demonstrated support for measurement invariance across groups for the SA-C model. The same procedure for testing measurement invariance was also applied for the C-A-S model [10], and this gave the same conclusion as for the SA-C model, i.e. support for measurement invariance across groups.

**Table 3:** *Tests for measurement invariance across the sample without alcohol problems and the sample with comorbid alcohol problems for the SA-C model.*

	*SB χ2*	*Df*	RMSEA	SRMR	CFI
*Beck et al. * [*7*] * SA-C:*					
Model 1: Configural model	623.2	372	.051	.050	.932
Model 2a: Factor loadings invariant	659.8	391	.051	.062	.927
Model 2b: Residual cov. Invariant	664.1	393	.051	.066	.926
Model 3: Item intercepts invariant	694.4	412	.051	.064	.923
Model 4: Item residual variance invariant	726.4	433	.051	.064	.920
Model 5: Factor variance invariant	729.8	435	.051	.072	.920
Model 6: Factor covariance invariant	731.0	436	.051	.071	.920

*SB χ^2^* = Satorra-Bentler corrected *χ^2^*; *df* = degrees of freedom; RMSEA = root mean square of approximation;

SRMR = Standardized root mean square; CFI = comparative fit index.

Table 4 shows the standardized factor loadings, factor correlations and error term correlations for the SA-C Beck model [7]. All parameters presented in Table 4 are significant at the 5% level.

**Table 4:** *Multigroup confirmatory factor analysis with robust maximum likelihood estimation. Standardized factor loadings, factor correlation and error correlations for the modified SA-C model* [*7*] *.*

	Standardized loadnings/correlations
**Cognitive**	
1 Sadness	.659
2 Pessimism	.607
3 Past Failure	.691
5 Guilty Feelings	.587
6 Punishment Feelings	.462
7 Self-Dislike	.679
8 Self-Criticalness	.663
9 Suicidal Thoughts or Wishes	.530
14 Worthlessness	.732
**Somatic-affective**	
11 Agitation	.403
15 Loss of Energy	.661
16 Changes in Sleeping Pattern	.394
17 Irritability	.418
18 Changes in Appetite	.511
19 Concentration Difficulty	.707
20 Tiredness or Fatigue	.616
21 Loss of Interest in Sex	.481
4 Loss of Pleasure	.718
10 Crying	.528
12 Loss of Interest	.706
13 Indecisiveness	.686
Correlation C–SA	.859
Correlation e5–e8	.340
Correlation e15–e20	.449

All loadings/correlations are significant at the .001 level.

### Differences in Level of Depression Between the Samples

To test whether the level of depression on the Somatic-Affective and the Cognitive factor was different in the two samples, MIMIC modeling was applied using group (without vs. with comorbid alcohol problems) as the independent variable, and gender and marital status as covariates (due to significant differences between groups on these two variables). The results of the analysis showed that the latent mean score was significantly higher on both the Somatic-Affective (0.185 (0.053), *p*<.001) and the Cognitive factor (0.175 (0.044), *p*<.001) for patients with comorbid alcohol problems compared to those without alcohol problems. These differences indicate that although the factor structure is very similar in the two samples, the level of depression varies.

In order to examine the magnitude of the difference in latent mean scores, differences between groups in terms of standard deviation on the latent variables was estimated by setting the variance in each factor equal to 1. The results showed that the relative differences between groups were of about the same size on both factors, i.e. 0. 418 and 0.348 standard deviation on the Cognitive factor and Somatic-Affective factor, respectively. According to the criteria suggested by Cohen [22], this represents a moderate effect size.

## Discussion

The results showed that the same models (i.e. Beck et al.'s [7] SA-C model and Buckley et al.'s [10] C-A-S model), gave the best fit to the data in both groups. The similarity in the factor structure of the BDI-II was further supported by the finding of measurement invariance across groups for both models. Although there are exceptions [see 13], this finding corroborates other studies examining samples of various drug abuse populations [10], [11], [12].

The two proposed factor structures are identical with regard to the Cognitive factor. The difference between the two models is that the Somatic-Affective factor in Beck et al.'s model is split into two factors in Buckley et al.'s [10] model: one Somatic factor and one Affective factor. Based on the small difference in model fit indices between the two models, one might argue that Beck et al.'s [7] SA-C model should be the preferred model due to its more parsimonious nature.

Patients with comorbid alcohol problems reported on average a higher degree of depressive symptoms on both the Somatic-Affective and the Cognitive factor. A higher score on depressive symptoms among patients with comorbid alcohol abuse has also described by Ostacher [4]. The magnitude of the difference in depressive symptoms on the two factors between the two groups was found to be of equal size, suggesting that alcohol abuse has an overall effect upon depressive symptoms. Others have claimed that the somatic factor in depression is closely related to abstinence and the intoxication of alcohol or other substances in patients with comorbid substance abuse [11]. On this basis, if is expected that the difference in level of depression between the two groups should primarily be found on the Somatic factor. This claim was not confirmed in our study [see also 12].

The results from the current study support the view that major depression and substance-induced depression differ mainly in the level of depressive symptoms, rather than in the structure of the symptoms [12]. However, it is difficult to determine the direction of causality between depressive symptoms and alcohol abuse. Our study indicates that depression-like symptoms in patients with alcohol problems are not merely transient, alcohol-induced effects. Patients with comorbid depression and alcohol abuse should be offered parallel and adequate treatment for both conditions.

There are some limitations in our study. The participants did not undergo a clinical interview to verify that their symptoms assessed with BDI-II indicated a formal diagnosis of depression. Despite these diagnostic limitations, we improved our understanding regarding the type and strength of symptoms in a sample of patients with depressive symptomatology. This study cannot illuminate the complex ways in which depressive symptoms and alcohol problems continue to interact over time. The strength is the size of the sample, which consists of two groups of depressed subjects with and without comorbid alcohol abuse.

### Conclusion

Although there were differences in the symptom load, the basic factor structure is similar in patients with depressive symptoms with and without unhealthy alcohol use. This finding may strengthen the arguments for giving both of these problems clinical attention during treatment.

## Supporting Information

Table S1 **Beck Depression Inventory-II.** Means, standard deviations and inter-item correlations for patients without alcohol problems (n = 358).
(DOC)

Table S2 **Beck Depression Inventory-II.** Means, standard deviations and inter-item correlations for patients with comorbid alcohol problems (n = 158).
(DOC)
