# Supplementary material for: Depressive Symptoms in People with and without Alcohol Abuse: Factor Structure and Measurement Invariance of the Beck Depression Inventory (BDI-II) Across Groups
Source: PLoS One. 2014 Feb 12;9(2):e88321. doi: 10.1371/journal.pone.0088321 (PMC3922762; doi:10.1371/journal.pone.0088321)
Supplement: Table S1 — Beck Depression Inventory-II. Means, standard deviations and inter-item correlations for patients without alcohol problems (n = 358). (DOC) [file pone.0088321.s001.doc]

*Table S1*. Beck Depression Inventory-II. Means, standard deviations and inter-item correlations for patients without alcohol problems (n = 358)

| *Item* | *M* | *SD* | *1* | *2* | *3* | *4* | *5* | *6* | *7* | *8* | *9* | *10* | *11* | *12* | *13* | *14* | *15* | *16* | *17* | *18* | *19* | *20* | *21* |
| --- | --- | --- | --- | --- | --- | --- | --- | --- | --- | --- | --- | --- | --- | --- | --- | --- | --- | --- | --- | --- | --- | --- | --- |
| 1 | 0.88 | 0.66 | - |  |  |  |  |  |  |  |  |  |  |  |  |  |  |  |  |  |  |  |  |
| 2 | 1.14 | 0.81 | .48 | - |  |  |  |  |  |  |  |  |  |  |  |  |  |  |  |  |  |  |  |
| 3 | 1.16 | 0.88 | .43 | .40 | - |  |  |  |  |  |  |  |  |  |  |  |  |  |  |  |  |  |  |
| 4 | 1.24 | 0.75 | .52 | .41 | .36 | - |  |  |  |  |  |  |  |  |  |  |  |  |  |  |  |  |  |
| 5 | 1.09 | 0.86 | .36 | .24 | .43 | .45 | - |  |  |  |  |  |  |  |  |  |  |  |  |  |  |  |  |
| 6 | 0.75 | 1.01 | .35 | .34 | .31 | .27 | .38 | - |  |  |  |  |  |  |  |  |  |  |  |  |  |  |  |
| 7 | 1.36 | 0.96 | .44 | .40 | .51 | .48 | .48 | .29 | - |  |  |  |  |  |  |  |  |  |  |  |  |  |  |
| 8 | 1.18 | 0.95 | .41 | .34 | .48 | .51 | .64 | .39 | .53 | - |  |  |  |  |  |  |  |  |  |  |  |  |  |
| 9 | 0.44 | 0.61 | .42 | .34 | .37 | .34 | .29 | .27 | .40 | .30 | - |  |  |  |  |  |  |  |  |  |  |  |  |
| 10 | 1.07 | 1.00 | .35 | .29 | .30 | .40 | .35 | .24 | .44 | .34 | .29 | - |  |  |  |  |  |  |  |  |  |  |  |
| 11 | 0.90 | 0.75 | .32 | .22 | .20 | .28 | .22 | .26 | .23 | .20 | .16 | .24 | - |  |  |  |  |  |  |  |  |  |  |
| 12 | 1.01 | 0.88 | .44 | .38 | .38 | .59 | .32 | .29 | .43 | .37 | .32 | .38 | .35 | - |  |  |  |  |  |  |  |  |  |
| 13 | 1.24 | 0.94 | .37 | .32 | .42 | .45 | .40 | .32 | .40 | .44 | .28 | .36 | .28 | .50 | - |  |  |  |  |  |  |  |  |
| 14 | 1.10 | 0.88 | .41 | .42 | .49 | .50 | .49 | .36 | .53 | .50 | .38 | .34 | .21 | .46 | .43 | - |  |  |  |  |  |  |  |
| 15 | 1.37 | 0.78 | .36 | .25 | .34 | .44 | .28 | .25 | .36 | .33 | .23 | .40 | .29 | .48 | .49 | .41 | - |  |  |  |  |  |  |
| 16 | 1.51 | 0.97 | .22 | .25 | .22 | .24 | .19 | .20 | .21 | .26 | .19 | .31 | .22 | .19 | .24 | .20 | .37 | - |  |  |  |  |  |
| 17 | 0.98 | 0.80 | .28 | .25 | .27 | .26 | .27 | .18 | .29 | .33 | .08 | .27 | .30 | .25 | .32 | .22 | .22 | .19 | - |  |  |  |  |
| 18 | 0.96 | 0.92 | .30 | .30 | .31 | .37 | .31 | .34 | .36 | .30 | .29 | .31 | .26 | .34 | .36 | .42 | .40 | .34 | .21 | - |  |  |  |
| 19 | 1.21 | 0.75 | .39 | .38 | .41 | .48 | .39 | .26 | .46 | .42 | .34 | .43 | .36 | .52 | .53 | .49 | .56 | .35 | .34 | .42 | - |  |  |
| 20 | 1.37 | 0.85 | .27 | .26 | .33 | .44 | .26 | .19 | .36 | .31 | .19 | .35 | .25 | .42 | .43 | .36 | .71 | .35 | .32 | .37 | .49 | - |  |
| 21 | 1.15 | 1.04 | .35 | .17 | .33 | .45 | .37 | .25 | .31 | .34 | .24 | .37 | .28 | .44 | .40 | .30 | .40 | .20 | .25 | .32 | .37 | .41 | - |
