# Supplementary material for: Depressive Symptoms in People with and without Alcohol Abuse: Factor Structure and Measurement Invariance of the Beck Depression Inventory (BDI-II) Across Groups
Source: PLoS One. 2014 Feb 12;9(2):e88321. doi: 10.1371/journal.pone.0088321 (PMC3922762; doi:10.1371/journal.pone.0088321)
Supplement: Table S2 — Beck Depression Inventory-II. Means, standard deviations and inter-item correlations for patients with comorbid alcohol problems (n = 158). (DOC) [file pone.0088321.s002.doc]

*Table S2*. Beck Depression Inventory-II. Means, standard deviations and inter-item correlations for patients with comorbid alcohol problems (n = 158)

| *Item* | *M* | *SD* | *1* | *2* | *3* | *4* | *5* | *6* | *7* | *8* | *9* | *10* | *11* | *12* | *13* | *14* | *15* | *16* | *17* | *18* | *19* | *20* | *21* |
| --- | --- | --- | --- | --- | --- | --- | --- | --- | --- | --- | --- | --- | --- | --- | --- | --- | --- | --- | --- | --- | --- | --- | --- |
| 1 | 1.12 | 0.64 | - |  |  |  |  |  |  |  |  |  |  |  |  |  |  |  |  |  |  |  |  |
| 2 | 1.30 | 0.83 | .50 | - |  |  |  |  |  |  |  |  |  |  |  |  |  |  |  |  |  |  |  |
| 3 | 1.44 | 0.90 | .43 | .45 | - |  |  |  |  |  |  |  |  |  |  |  |  |  |  |  |  |  |  |
| 4 | 1.39 | 0.77 | .57 | .47 | .45 | - |  |  |  |  |  |  |  |  |  |  |  |  |  |  |  |  |  |
| 5 | 1.24 | 0.77 | .31 | .34 | .36 | .34 | - |  |  |  |  |  |  |  |  |  |  |  |  |  |  |  |  |
| 6 | 1.01 | 1.13 | .16 | .15 | .33 | .23 | .31 | - |  |  |  |  |  |  |  |  |  |  |  |  |  |  |  |
| 7 | 1.55 | 0.93 | .30 | .41 | .43 | .30 | .29 | .09 | - |  |  |  |  |  |  |  |  |  |  |  |  |  |  |
| 8 | 1.33 | 0.86 | .39 | .36 | .44 | .38 | .49 | .31 | .49 | - |  |  |  |  |  |  |  |  |  |  |  |  |  |
| 9 | 0.68 | 0.63 | .38 | .35 | .34 | .32 | .19 | .15 | .36 | .25 | - |  |  |  |  |  |  |  |  |  |  |  |  |
| 10 | 1.19 | 1.12 | .39 | .34 | .34 | .30 | .22 | .27 | .36 | .35 | .39 | - |  |  |  |  |  |  |  |  |  |  |  |
| 11 | 1.07 | 0.74 | .18 | .15 | .12 | .12 | .37 | .15 | .05 | .27 | -.02 | .04 | - |  |  |  |  |  |  |  |  |  |  |
| 12 | 1.25 | 0.86 | .40 | .52 | .48 | .58 | .36 | .21 | .25 | .28 | .19 | .21 | .16 | - |  |  |  |  |  |  |  |  |  |
| 13 | 1.46 | 0.96 | .43 | .46 | .53 | .42 | .45 | .26 | .33 | .47 | .19 | .29 | .32 | .52 | - |  |  |  |  |  |  |  |  |
| 14 | 1.24 | 0.88 | .46 | .50 | .66 | .44 | .31 | .35 | .50 | .50 | .42 | .31 | .06 | .50 | .47 | - |  |  |  |  |  |  |  |
| 15 | 1.53 | 0.70 | .42 | .39 | .37 | .52 | .28 | .10 | .38 | .25 | .25 | .24 | .17 | .53 | .44 | .41 | - |  |  |  |  |  |  |
| 16 | 1.70 | 0.89 | .21 | .16 | .07 | .28 | .14 | -.03 | .17 | .26 | .08 | .09 | .18 | .15 | .15 | .15 | .24 | - |  |  |  |  |  |
| 17 | 1.00 | 0.83 | .26 | .17 | .19 | .35 | .25 | .19 | .21 | .29 | .05 | .14 | .20 | .27 | .23 | .26 | .24 | .31 | - |  |  |  |  |
| 18 | 1.17 | 0.93 | .27 | .20 | .20 | .24 | .33 | .31 | .22 | .33 | .20 | .26 | .12 | .11 | .24 | .23 | .22 | .20 | .22 | - |  |  |  |
| 19 | 1.32 | 0.69 | .37 | .39 | .34 | .43 | .36 | .20 | .28 | .30 | .25 | .22 | .27 | .44 | .50 | .32 | .37 | .15 | .22 | .18 | - |  |  |
| 20 | 1.53 | 0.88 | .33 | .32 | .32 | .44 | .34 | .15 | .37 | .30 | .18 | .24 | .18 | .38 | .40 | .34 | .60 | .38 | .33 | .32 | .45 | - |  |
| 21 | 1.06 | 0.96 | .24 | .23 | .10 | .26 | .18 | .07 | .18 | .16 | .13 | .13 | .00 | .17 | .13 | .13 | .10 | .18 | .12 | .30 | .19 | .19 | - |
